# Supplementary material for: Clinical Characteristics and Prognostic Factors Affecting Clinical Outcomes in Cytomegalovirus Retinitis Following Allogeneic Hematopoietic Stem Cell Transplantation
Source: Biomedicines. 2025 Jan 20;13(1):242. doi: 10.3390/biomedicines13010242 (PMC11760473; doi:10.3390/biomedicines13010242)
Supplement: Supplementary file 1 [file biomedicines-13-00242-s001.zip › Supplementary Figure S1 and Table S1.pdf]

Figure S1: Difference in rates of retinal detachment (A) and recurrence (B) between improved/stabilized group and deterioration group

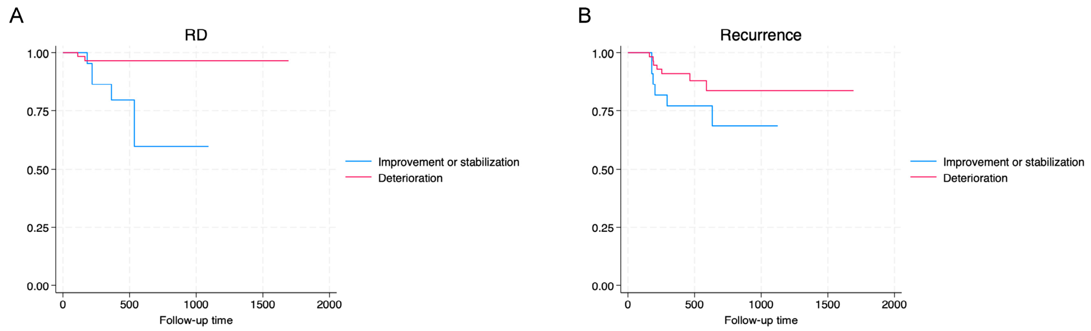

Supplementary Table S1 Demographic characteristics of patients with bilateral or unilateral involvement.

	Unilateral	Bilateral	P-value
Age, median (IQR)	28 (21.5, 30.5)	27.5 (21, 35)	0.889
Female: male	11:13	11:19	0.496
HLA match: mismatch	1:23	4:26	0.248
Donor related: unrelated	24:0	26:4	0.063
ABO match: mismatch	7:17	17:13	0.043*
Poor graft function, n (%)	5(20.8)	9(30)	0.445
GVHD, n(%)	19 (79.2)	19 (63.3)	0.205
Other organ involvement with CMV infection, n (%)	2(8.3)	7 (23.3)	0.142
PTLD, n (%)	1 (4.2)	6 (20)	0.085
Duration of lymphopenia, median (IQR)	63.5 (49, 107)	64 (46, 116)	0.955
EBV DNAemia, n (%)	9 (40.9)	13 (48.2)	0.612

*P<0.05. IQR, interquartile range; HLA, human leukocyte antigen; GVHD, graft-versus-host disease; CMV, cytomegalovirus; PTLD, post-lymphoproliferative disease; EBV, Epstein-Barr virus.
